# Emerging Infectious Diseases: 10 Years Running

**DOI:** 10.3201/eid1103.050191

**Published:** 2005-03

**Authors:** Joseph E. McDade, Polyxeni Potter, D. Peter Drotman

**Affiliations:** * Centers for Disease Control and Prevention, Atlanta, Georgia, USA

**Keywords:** Emerging Infectious Diseases, electronic publishing, 10th anniversary, commentary, editorial

With this volume, Emerging Infectious Diseases (EID) turns 10—a time probably between childhood and adolescence in journal years. All of us associated with the journal are very proud of this milestone, but at the same time we are aware of the need to continually assess progress toward original goals and make changes when and where needed.

EID’s genesis stems from advice to the Centers for Disease Control and Prevention (CDC) promulgated by the 1992 Institute of Medicine report, Emerging Infections: Microbial Threats to Health in the United States (*1*). EID was envisaged as a vital component of CDC’s effort to communicate the threat of emerging infections worldwide. Fully and for the most part externally peer reviewed, the journal would have an international editorial board and would seek a global readership base. Its broadbased content would mirror the complex microbial, demographic, genetic, economic, technologic, behavioral, social, and other factors (including nefarious ones) that contribute to infectious disease emergence. The format would include views and summaries of major disease topics and trends, reports of new agents, reservoirs or foci of infection, and timely or preliminary results of relevant research. To make possible swift global dissemination of this compelling content, the journal would be fully electronic and be distributed free of charge, a concept being robustly discussed in 2005 by many editors and publishers under the topic of “open access.” Special features were soon added to emphasize the links between science, public health, and the human condition.

If submissions and subscriptions are a measure of reader interest, Emerging Infectious Diseases has enjoyed some success during its first decade. Readership (infectious disease professionals in academia, clinical practice, industry, public health, and related disciplines) has grown from 3,000 subscribers in 1995 to >45,000 (print and online, >6,000 international). Submissions are increasing and number >120 per month. Although most submissions come from scientists in the United States and Western Europe, manuscripts continue to arrive in substantial and increasing numbers from every part of the globe. The journal, which is widely indexed, has a consistently high impact factor (fourth most cited of 41 infectious disease journals, Institute of Scientific Information citation reports, 2003).

Electronic publishing, the dynamic concept driving Emerging Infectious Diseases, has made huge strides during the journal’s first decade. Ahead-of-print publication, a founding feature of Emerging Infectious Diseases, is now common practice in the scientific journal community. Online-only publication of technical and lengthy but valuable parts of articles has also been adopted by many journals. Multiple links to references, databases, and other relevant information have become standard. Interactive features and instant access to authors and editors have revolutionized scientific dialog. EID’s impulse to swiftly provide urgent information free of charge to a global audience has been reaffirmed by several recent movements toward free access to scientific data.

During its 10-year journey, Emerging Infectious Diseases has had some advantages. Providing unrestricted access to all content has allowed the journal to reach its intended audience of public health professionals around the globe. In-house production has made possible timely posting of articles on the Web as soon as they are cleared for publication. A small but uniquely competent and flexible production team, dedicated associate editors and editorial board, and thousands of volunteer expert reviewers have energized our journal’s growing contribution to the public health community.

Like all anniversaries, our journal’s 10th is a combination of reflecting on the past and looking into the future. From the public health perspective, the world’s current situation is every bit as volatile as it was 10 years ago, perhaps even more so. New infectious diseases and etiologic agents (e.g., avian influenza, Nipah virus infection, SARS) continue to surface relentlessly. Other diseases are finding new niches. West Nile virus encephalitis, endemic to the Eastern Hemisphere, became established in the northeastern United States in 1999 and continues to spread each year. As predicted within the public health community, multidrug-resistant bacterial infections have become increasingly commonplace, yet development of new antimicrobial drugs has failed to keep pace. Against this backdrop of naturally occurring problems, intentional release of pathogenic organisms has surfaced as a threat to public health and global security. The Institute of Medicine revisited the issue they brought to the world’s attention in 1992 with another report in 2003 (*2*). Their advice is not dissimilar to that of the Red Queen to Lewis Carroll’s Alice in Wonderland: “…it takes all the running you can do, to keep in the same place. If you want to get somewhere else, you must run at least twice as fast as that!” (*3*).

From the journal publishing perspective, continued emergence of new diseases and agents compels rapid dissemination of relevant scientific and public health information on the Web. As electronic access has become more commonplace, Web audiences have increased, and while in 1995 we had to educate readers on how to access our electronic journal, now we strive to keep up with their increased sophistication and need for additional research tools. In the ever-changing world of electronic publishing, speed is no longer the greatest challenge. Traditional publishing bottlenecks are diminishing. Web-based submission and peer review shrink the time from manuscript submission to publication. Online editing and production have advanced to the benefit of the final product. The question is no longer how fast information can be published but how content quality can keep up with technologic speed (*4*). When anthrax spores were disseminated through the U.S. postal system, Emerging Infectious Diseases published online a peer-reviewed article describing the first 10 cases. This definitive article (*5*), published online 5 days after submission, provided healthcare professionals the information needed to recognize potential new cases. When SARS burst onto the public health scene, Emerging Infectious Diseases published an entire issue with >40 peer-reviewed articles on a disease that 12 months earlier was not known to exist (see http://wwwnc.cdc.gov/eid/content/10/2/contents.htm). The technology that allowed these and other cases of expedited publication continues to advance. However, the ability to collect and interpret data on unknown diseases and uncharacterized agents follows a different timetable, in which quality must continue to take precedence over speed.

Electronic publishing provides solutions and poses challenges. Online-only publication for selected sections of journal materials may, in the short term, ease space constraints that threaten the journal’s ability to remain inclusive. Expansive indexing will further refine electronic search capabilities, increase links to large electronic databases, and improve the pace and quality of emerging infections and other research. The breadth and complexity of online activities will continue to demand increased technical expertise and exceptional flexibility from all involved with the journal—authors, reviewers, editors, and production staff.

As Emerging Infectious Diseases begins its second decade, its primary goal remains to communicate the threat of emerging infections worldwide and reduce the global impact of these infections, particularly among the young, the old, and the immunocompromised. The principal difference between 1995 and now is the pace, both of current events and of expected publication. Our challenge for the coming decade is not only to keep up with an increasing pace, but also to set the pace whenever we can, or in the words of one track coach, “Start off as fast as you can, and then gradually pick up speed.”
